# Efficacy of Platelet-Rich Plasma Containing Xenogenic Adipose Tissue-Derived Stromal Cells on Restoring Intervertebral Disc Degeneration: A Preclinical Study in a Rabbit Model

**DOI:** 10.1155/2019/6372356

**Published:** 2019-04-16

**Authors:** Chao Ma, Ran Wang, Dingliang Zhao, Naikun Wang, Ying Han, Shichong Wang, Tianyun Gao, Bin Wang, Lijuan Lu

**Affiliations:** ^1^Department of Pain, The Drum Tower Hospital Affiliated to Medical School of Nanjing University, No. 321 Zhongshan Road, Nanjing 210008, Jiangsu Province, China; ^2^Center for Clinic Stem Cell Research, The Drum Tower Hospital Affiliated to Medical School of Nanjing University, No. 321 Zhongshan Road, Nanjing 210008, Jiangsu Province, China

## Abstract

**Objective:**

Platelet-rich plasma (PRP) containing multiple growth factors is a promising strategy for disc degeneration. Thus, this study hypothesizes that the combination of PRP and adipose tissue-derived stromal cells (ADSCs) may repair degenerative disc more effectively than using each one of them alone.

**Methods:**

The model of early intervertebral disc degeneration was induced by annular puncture in the New Zealand rabbit. Autologous PRP was extracted from fresh arterial blood by using two centrifugation techniques. ADSC was offered by the Center for Clinic Stem Cell Research. Four weeks after the first experiment, PRP or ADSCs or a combination of PRP and ADSCs was injected into the punctured intervertebral disc. Four weeks later, disc height and signal intensity on T2-weighted magnetic resonance imaging (MRI) were assessed.

**Results:**

One month after puncture, we detected relatively narrow discs and lower signal intensity in MRI T2-weighted images. At four weeks after injection, the PRP-ADSC group statistically significantly restored discs, compared with PRP, ADSCs, or negative control group.

**Conclusions:**

The combination of PRP and ADSCs shows an effective potential to restore degenerated intervertebral discs in the rabbit.

## 1. Introduction

Intervertebral disc degeneration (IDD) is the leading cause of low back pain (LBP), leading to disability and placing a heavy burden on society [1]. Current treatment of IDD, such as conservative procedures (medication or physical therapy) and operative treatment, only partly alleviates symptoms, but does not reverse intervertebral disc cataplasia in etiology or pathology [2]. As a consequence, how to augment the number and enhance the function of nucleus pulposus cells has become a research hotspot [3].

Including PDGF, TGF-*β*, VEGF, and so on, PRP contains various growth factors that promote cell proliferation and tissue regeneration [4]. At the same time, PRP regulates the extracellular microenvironment by inducing extracellular matrix synthesis and secretion [5]. Furthermore, the role of PRP in immune inflammation is the production of interleukins and chemokines [6, 7]. PRP has been proven to be an effective therapy for IDD in rats, rabbits, and sheep treated in a large number of experimental animal models [2, 8]. Compared to other animals, rabbits are cheaper and their intervertebral discs are relatively large. Clinical research studies in the field of IDD have been indicative that treatment with PRP has a pronounced effect [9, 10].

Stem cell therapy is gradually becoming popular in multiple fields including IDD [11]. ADSCs have biological advantages in the proliferative capacity, secreted proteins, and immunomodulatory effects [12]. After passing through the needle at constant flow rates, ADSCs maintain proliferative capacity and metabolic function [13]. Scientists verify favorable safety profile of ADSCs in a rabbit model for osteoarthritis [14]. In our current study, we hypothesized that the combination of PRP and ADSCs may possess synergistic restorative function on the degenerative disc, considering ADSCs secreting protein to enhancing the function of PRP. The purpose of this study was to investigate the effects of ADSCs containing PRP on the regeneration of early degenerated intervertebral discs in vivo. To achieve this aim, MRI evaluations were utilized to detect differences in treated discs.

## 2. Materials and Methods

### 2.1. ADSC Isolation and Identification

This study was approved by the Research Ethics Board of Nanjing Drum Tower Hospital. Written consent was obtained from the healthy adipose tissue donors. ADSCs were prepared by following the method in GMP (good manufacture practice) facility. After washing with phosphate-buffered saline (PBS), the adipose tissue was cut into small pieces of approximately 1 mm^3^ and was digested with 0.075% collagenase (type I; Sigma-Aldrich, St. Louis, MO, USA) at 37°C for 30 min. The digestion was stopped by adding *α*-MEM containing 10% FBS, and the solution was centrifuged at 1200 × *g* for 10 min. The cell pellet was resuspended and filtered through a 100 *μ*m Nylon mesh to remove tissue remains. The cells were incubated in the culture medium and were passaged three times to remove the contamination of other types of cells. ADSCs at the fourth passage were used for all of the experiments in this study.

Flow cytometry analysis was used for phenotypic analysis in ADSCs. A total of 100,000 cells at the fourth passage were incubated with fluorescein isothiocyanate (FITC) or phycoerythrin (PE) labeled monoclonal antibodies (CD11b FITC, CD34 FITC, CD44 PE, CD45 FITC, CD73 PE, CD105 PE, HLA-DQ FITC, and HLA-DR FITC; BD, San Diego, CA, USA) for 30 min in the dark at room temperature. ADSCs were centrifuged at 2000 rpm for 5 min after washing three times with 1 × PBS and resuspended in 1 × PBS for flow cytometry analysis. Cells were analyzed using a FACScan (BD FACSAria™; BD, San Jose, CA, USA), and data were analyzed with the FACS software.

ADSCs were cultured in a 24-well tissue culture plate at a density of 1 × 10 exp 4 cells/well for adipogenic and osteogenic differentiation. ADSCs were cultured with adipogenic differentiation medium, osteogenic differentiation medium, and cartilage differentiation medium (Gibco, Grand Island, NY, USA) at 50–70% confluency. Every 3-4 days, the differentiation medium was changed. At day 21, Oil Red O staining (Sigma-Aldrich, St. Louis, MO, USA), Alizarin Red-S staining (Sigma-Aldrich, St. Louis, MO, USA), and Alcian blue staining (Sigma-Aldrich, St. Louis, MO, USA) were performed to check the adipogenesis, osteogenesis, and cartilage differentiation potentials of ADSCs. The cells used in this study come from a single donor, and the quality was fully evaluated to meet the requirements and standards of MSC's quality and safety for clinic settings.

### 2.2. Experimental Animals

A total of five rabbits (female, 2.5–3.5 kg, 4–6 weeks) were grown in the animal lab of Drum Tower Hospital Affiliated to Medical School of Nanjing University. The breed of rabbits was a New Zealand white rabbit. As the narrow area of our animal laboratory, our team had only five cages. To take full advantage of these rabbits, we established four degenerative discs (L2/3, L3/4, L4/5, and L5/6) in each rabbit under the general anesthesia by intraperitoneal injection of 10% chloral hydrate. The discs were divided into five groups (Group A: L1/2 discs were set as normal control group; Group B: L2/3 discs were set as degeneration and PBS injection; Group C: L3/4 discs were set as degeneration and PRP injection; Group D: L4/5 discs were set as degeneration and ADSC injection; and Group E: L5/6 discs were set as degeneration and combined ADSC and PRP injection). Our study design was approved by the Animal Ethics Committee of Drum Tower Hospital of Nanjing University.

### 2.3. Establishment of IDD Model and Evaluation of MR

Preoperatively, rabbits were accepted intraperitoneal injection with 10% chloral hydrate. The height of the intervertebral disc was measured by magnetic resonance (MR) as the base line prior to puncture. Then, we used a sterile towel to remove the fur, disinfect the skin, and cover the rabbit. The IDD model of rabbits was established by repeatedly puncture the annulus fibrosus of the intervertebral using the puncture needle (22G) under CT. Ct-guided disc puncture did not require an incision into the rabbit's posterior back, reducing bleeding and infection. The nucleus pulposus and annulus fibrosus of the intervertebral disc were broken by puncture needle (22G) and negative pressure suction (the empty needle piston scale ranged from 0 ml to 2 ml for 15 seconds) can destroy the nucleus pulposus and induce degeneration model. To confirm the IDD model and evaluate the effect of biology therapy, we used MR to scan the target disc until 4 weeks after the first experiment and injection of biological agents, respectively.

MRI was performed using a 1.5 T imager unit. Following general anesthesia with chloral hydrate, the rabbits were placed in a prone position for MRI scan. The signal intensity of the discs was evaluated by using T2-weighted images. IDD grade was evaluated using the modified Thompson classification of Grade I–IV. Two doctors evaluated the images separately. The parameters of the T2-weighted images wereas follows: time-to-repeat = 1,800 ms; field of view = 140 × 140 mm; time-to-echo = 70 ms; slice thickness = 2 mm; image matrix = 286 × 385; averages = 9. All the MRI signal scores were detected by the software of Image J.

### 2.4. Preparation of PRP, ADSCs, and PRP-ADSC Mixture

PRP was prepared by the methods of Aghaloo [15]. The rabbits were fixed on the operating table and arterial blood was drawn from the auricular central artery. After mixing with sodium citrate using as anticoagulant, the arterial blood was centrifuged in a two-step process. The first centrifugation was carried out at a rate of 215 g for 10 min, and the plasma above the white film was placed in another centrifuge tube after centrifugation. The second centrifugation was carried out at a speed of 863 g for 6 min. The supernatant, or platelet-poor plasma, was collected and transferred to another centrifuge tube. The remaining platelet-rich plasma was blown evenly. In our study, platelet-rich plasma contained small amounts of white blood cells. And we made sure that the count of platelet was greater than 1000 ∗ 10^9^/L (the count of platelets was 1041 ∗ 10^9^/L in our study) under the microscope.

**Figure 1:**
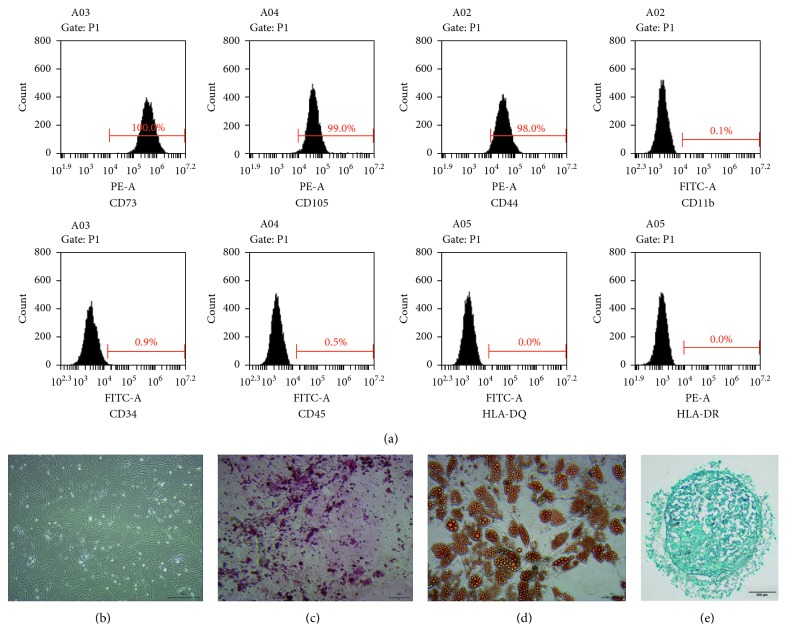
The cells were identified as ADSCs. (a) Surface markers of ADSCs were assayed by flow cytometry analysis. (b–e) The adipogenic, osteogenic, and cartilage differentiations of ADSCs were assayed by Oil Red O staining, Alizarin red-S staining, and Alcian blue staining.

The ADSCs were afforded by the Center for Clinic Stem Cell Research, the Affiliated Nanjing Drum Tower Hospital of Nanjing University Medical School (Figure 1). The number of stem cells in each group was ten million. A mixture of PRP, ADSCs, and PRP-ADSCs was included, each having a volume of 200 *μ*l. The puncture needle that injected the stem cells or PRP or PBS (phosphate-buffered solution) into the intervertebral disc was 22G.

### 2.5. Statistical Analysis

Statistical analysis was implemented by using the SPSS 18.0 software. Data of all groups are presented as mean ± standard deviation and were analyzed using variance analysis. The data of the MRI image signal were analyzed using a one way ANOVA. All the data were made by the software Graph Pad Prism. *P* < 0.05 was regarded as statistically significant difference.

## 3. Results

### 3.1. IDD Model of Rabbits Was Confirmed Successfully

**Figure 2:**
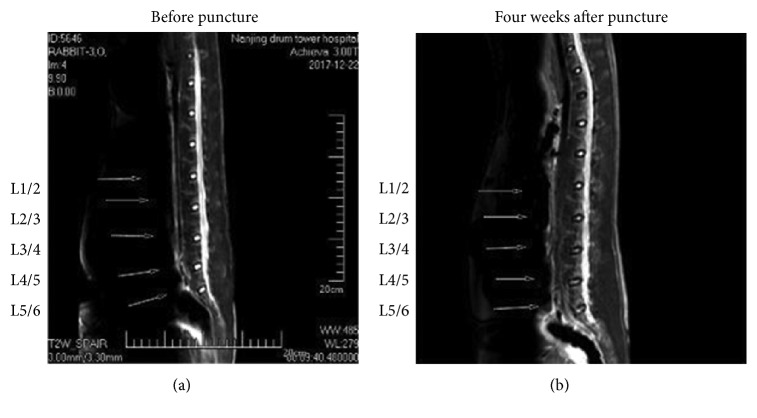
The signal of intervertebral discs (L2/3, L3/4, L4/5, and L5/6) in magnetic resonance (MR) T2-weighted images appeared to be significantly lower in (b) compared with (a), while in intervertebral discs L1/2, magnetic resonance images showed no difference on T2-weighted images.

**Figure 3:**
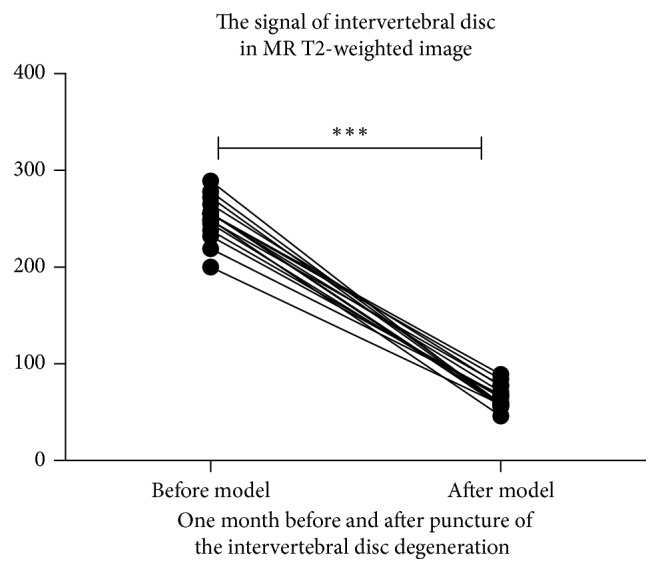
On the 28th day (4 weeks) after model establishment, the signal strength of degeneration disc detected by MRI was significantly lower than that detected before the model group (*p* < 0.001). ^*∗*^*p* < 0.05; ^*∗∗*^*p* < 0.01; ^*∗∗∗*^*p* < 0.001.

After 4 weeks of the first puncture, the signal images of discs included L2/3, L3/4, L4/5, and L5/6 were significantly lower than baseline as well as the control group (L1/2). This means that our annulus fibrosus punctures lead to the degenerative disc (Figures 2 and 3).

### 3.2. Either of PRP, ADSCs, or PRP-ADSC Mixture Could Restore Degenerative Disc

**Figure 4:**
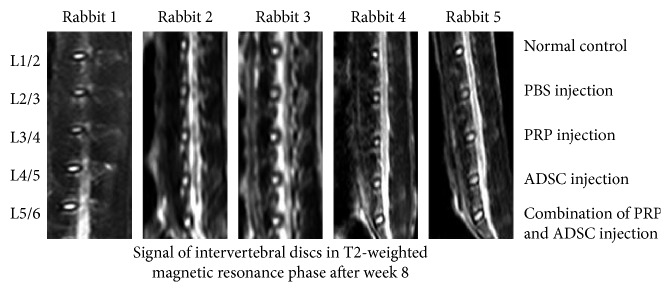
The injection of stem cells combined with platelet-rich plasma can significantly reverse the disc degeneration. The disc signals in magnetic resonance T2-weighted image in the group of ADSC-combined PRP showed higher signal strength than the other groups.

**Figure 5:**
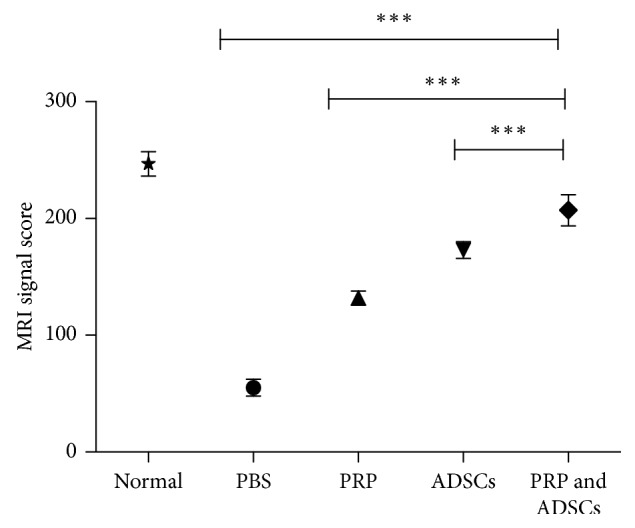
Compared with the negative control group, signal strength of the group of PRP, ADSCs, and PRP combination with ADSCs was remarkably high. Most important of all, PRP combination with ADSCs has the best effect in reversing degeneration.

Compared with disc L2/3, the signal image of L3/4, L4/5, and L5/6 were significantly higher. These results suggest that biological therapy including but not limited to PRP and ADSCs could promote the repair of disc regression. PRP-combined ADSCs had the most effect in restoring the intervertebral disc (Figures 4 and 5 and Table 1).

## 4. Discussion

This present study investigated the efficacy of PRP-containing ADSCs on degenerative disc regeneration induced by needle puncture. The results of this study demonstrated that the injection of PRP-containing ADSCs was effective in restoring the early degenerated discs. The MRI examination revealed the repair effects of the PRP-ADSCs on the early degenerated discs.

In the United States and the European Union, there was an increasing prevalence of the PRP utilization to facilitate healing in a variety of diseases, including musculoskeletal injuries, low back pain, and so on [16, 17]. Currently, PRP injection is frequently used in the clinical treatment of knee osteoarthritis [18, 19]. In the intervertebral disc degeneration, PRP treatment concentrates more on animal research or preliminary clinical trials, and more clinical randomized control trail (RCT) studies are still needed. In Spain, Japan, and American, retrospective single-center analysis has demonstrated that injection of autologous PRP was safe in patients with low back pain without adverse events [17, 20, 21].

Based on the reported animal research, the use of stem cells for the treatment of intervertebral disc degeneration is safe and effective. The incidence of complications in mesenchymal stem cell therapy for disc degeneration is low [22]. From 2010 to 2011, two literatures reported that intervertebral disc degeneration therapy using mesenchymal cell transplantation brought favorable results in patients diagnosed with lumber disc degeneration and have been followed up for 1 or 2 years [23, 24]. A randomized controlled trial concerning allogeneic mesenchymal bone marrow cells used in intervertebral disc repair displayed rapid and significant improvement [25].

The joint use of PRP and stem cells in patients with partial tear of the rotator cuff tendon alleviated pain and improved shoulder function [26]. Scientists combined PRP and bone marrow-derived mesenchymal stem cells to treat a model of disc degeneration caused by annular puncture in rabbit [27]. Literature studies manifested PRP can stimulate proliferation and differentiation of adipose tissue-derived mesenchymal stem cells by releasing diverse growth factors [28, 29]. Although our team firstly applied ADSC and PRP to disc degeneration, there is still a long way to go in regenerative medicine. More in-depth foundational and clinical research is indispensable.

There are some drawbacks in our work. However, there are still quite a few scientists using rabbits as models for disc degeneration as in 2018. We have to admit that the persistence of nucleus pulposus cells in rabbit models may be affected by notochord cells. We all know that it is better to do stem cell transplantation in immune-deficient mice, but the intervertebral discs of mice are too small and the intervertebral disc puncture is more difficult, which can lead to bleeding and death of animals. Due to the limitations of the animal room, the hospital only gave us a few rabbit cages. We only had 5 rabbits in this trial, but we used the intervertebral discs of rabbits as far as possible. After all, the results suggest that the combination of PRP and ADSC transplantation can be safe and effective in the treatment of degenerative disc induced by annular puncture in the New Zealand rabbit and encourage more studies with a larger sample. Next, we will expand the sample size and compare the difference between bone marrow mesenchymal stem cells and adipose stem cells in the collaborative PRP to reverse disc degeneration.

## Figures and Tables

**Table 1:** The signal intensity of the discs was evaluated by using T2-weighted images. All the MRI signal scores in five groups were detected by the software Image J.

Group	MRI signal score
Normal	246.8 ± 10.4
PBS	55.2 ± 7.3
PRP	131.8 ± 6.0
ADSCs	173 ± 7.1
PRP-combined ADSC injection	207 ± 13.5

## Data Availability

The data used to support the findings of this study are available from the corresponding author upon request.

## Abbreviations

ADSCs: Adipose tissue-derived stromal cells
PRP: Platelet-rich plasma
MRI: Magnetic resonance imaging
IDD: Intervertebral disc degeneration
LBP: Low back pain
BMSCs: Bone marrow-derived mesenchymal stem cells
MR: Magnetic resonance.
